# Neutrophil CD64–a prognostic marker of sepsis in intensive care unit: a prospective cohort study

**DOI:** 10.3389/fmed.2023.1251221

**Published:** 2023-09-08

**Authors:** Huy Minh Pham, Duy Ly Minh Nguyen, Minh Cuong Duong, Xuan Thi Phan, Linh Thanh Tran, Duong Hong Thuy Trang, Thao Thi Ngoc Pham

**Affiliations:** ^1^Department of Emergency and Critical Care, Faculty of Medicine, University of Medicine and Pharmacy at Ho Chi Minh City, Ho Chi Minh City, Vietnam; ^2^Intensive Care Unit, Cho Ray Hospital, Ho Chi Minh City, Vietnam; ^3^School of Population Health, University of New South Wales, Sydney, NSW, Australia; ^4^Intensive Care Unit, Nguyen Trai Hospital, Ho Chi Minh City, Vietnam

**Keywords:** nCD64, sepsis, prognosis, intensive care unit, ICU

## Abstract

**Background:**

Little is known about the prognostic ability of nCD64 in critically ill patients. This study aimed to assess the prognostic values of nCD64 in adult ICU patients with sepsis.

**Methods:**

A prospective cohort study was conducted at the ICU of Cho Ray Hospital in Vietnam between January 2019 to September 2020. All newly admitted 86 septic patients diagnosed based on sepsis-3 criteria were included. An evaluation of nCD64 was performed at admission (T0) and 48 h thereafter (T48). Delta nCD64 (nCD64 T48 – nCD64 T0), %delta nCD64 [(nCD64 T48 – nCD64 T0)/nCD64 T0 x 100%], APACHE II and SOFA scores were calculated and examined. Serum procalcitonin levels and white blood cell counts were documented. Spearman’s rank correlation coefficient was used to test the correlation between nCD64 and severity scores. Receiver-operating characteristic (ROC) curve was performed to evaluate the predictive efficacy of the sepsis parameters.

**Results:**

Patients with septic shock had significantly higher nCD64 levels than septic patients [3,568 (2,589; 5,999) vs. 1,514 (1,416;2,542) molecules/cell, *p* < 0.001]. nCD64 T0 and SOFA scores had a moderately positive linear correlation (*R* = 0.31, *p* = 0.004). In the survivor group, nCD64 levels significantly decreased within the first 48 h of admission (*p* < 0.001), while this trend was not statistically significant in the non-survivor group (*p* = 0.866). The area under the ROC curve (AUC) value of %delta nCD64 combined with APACHE II score (0.81) was higher than that of any other parameter alone or in combination with each other.

**Conclusion:**

The nCD64 index may serve as a valuable biomarker for predicting the course of sepsis. Monitoring changes in nCD64 during the initial 48 h of admission can aid in predicting the prognosis of septic patients. The use of a combination of the trends of nCD64 index in the first 48 h with APACHE II score would further enhance the predictive accuracy. More studies with longer follow-ups are needed to fully understand the implications of serial trend and kinetics of nCD64 in septic patients.

## Background

Sepsis is a condition in which a life-threatening organ dysfunction occurs due to the dysregulated host response to infection (1). Although sepsis is a major public health problem worldwide, the global impact of sepsis is difficult to determine because of the various, non-comparable methods used to quantify data in related studies (2). However, according to an analysis for the Global Burden of Disease Study in 2017, there were 48.9 million patients with sepsis and 11 million related deaths globally, accounting for approximately 20% of all global deaths (2). In a large meta-analysis of 170 studies conducted in Europe, North America, and Australia, the sub-analysis of 25 studies between 2009 and 2017 and 37 studies between 2011 and 2019 found that the 90-day mortality of sepsis and septic shock was 32.2 and 38.5%, respectively (3). Available data from Asian countries (including Vietnam) suggest that the overall prevalence of sepsis in intensive care units (ICUs) was 22.4%, and the in-hospital mortality rate of sepsis was 32.6% (4). It has been well documented that the prognosis and mortality of patients with sepsis are strongly influenced by the early detection of this health condition and timely treatment (5–7).

To improve outcomes of patients with sepsis, a reliable predictor of mortality and morbidity of this condition which helps monitor disease progression and guide timely treatment is needed (8). Indeed, several studies have been conducted to identify markers for the early identification and prognosis of sepsis (9–11). More recently, it has been found that the neutrophil cluster of differentiation 64 (nCD64) which is also known as the high-affinity immunoglobulin Fc-receptor I (FcγR1) (12) is a sensitive and specific marker for diagnosing sepsis caused by bacterial infections and for distinguishing sepsis from non-septic conditions (13–15). Studies comparing nCD64 with C reactive protein (CRP), procalcitonin (PCT), and other common biomarkers of sepsis including white blood cell (WBC) count and interleukin-6 (IL-6) have found a superior performance of nCD64 in early detecting sepsis (14, 16, 17). However, the use of nCD64 as a prognostic marker for sepsis in critically ill patients in ICU settings remains controversial (18). Studies have found that septic patients with a high nCD64 index at ICU admission have a higher survival rate (19, 20), while others have reported that higher levels of nCD64 are associated with poorer outcomes (21, 22). It should also be noted that most existing studies about nCD64 as a predictive marker in sepsis have exclusively examined a single value of nCD64 at admission, and a few studies have evaluated the prognostic usefulness of the changes of this biomarker over time (18). In addition, given that the definition of sepsis is shifted over time (23), none of these studies concurrently examined the dynamic of nCD64 as a prognostic marker of sepsis and utilized the most current definition of sepsis - sepsis-3 criteria (1). The presenting study aimed to examine the reliability and dynamic of the nCD64 index as a prognostic marker for ICU mortality in adult patients with sepsis diagnosed based on sepsis-3 criteria by comparing the sensitivity, specificity and area under the receiver operating characteristic (ROC) curve (AUC) of nCD64 with those of the commonly used morbidity severity score and mortality estimation tools including the Sequential Organ Failure Assessment (SOFA) score and Acute Physiology and Chronic Health Evaluation (APACHE) II score (11, 24, 25). The secondary outcomes included ventilator days, ICU and hospital length of stay.

## Methods

### Study design and setting

A prospective cohort study was conducted at the General ICU of Cho Ray Hospital (CRH) in Ho Chi Minh City, Vietnam from January 2019 to October 2020. CRH with over 2,300 beds is the largest tertiary referral hospital located in the southern Vietnam (26). The General ICU which is among the four ICUs of CRH, has 28 beds and receives critically ill patients from the Emergency Department of CRH and other hospitals in the region. Based on our experience, the majority of patients at the General ICU are those with multitrauma, sepsis, or septic shock.

During the study period, all septic patients who had a clear source of infection and met the diagnostic criteria for sepsis in accordance with the third International Consensus Definitions for Sepsis and Septic Shock (Sepsis-3) (1) were invited to participate in the study. In detail, sepsis is defined as a life-threatening organ dysfunction caused by a dysregulated host response to infection (1). Organ dysfunction can be identified as an acute change in total SOFA score ≥ 2 points consequent to the infection (1). Patients with septic shock can be identified with a clinical construct of sepsis with persisting hypotension requiring vasopressors to maintain the mean arterial pressure (MAP) ≥65 mmHg and having a serum lactate level > 2 mmol/L (18 mg/dL) despite adequate volume resuscitation (1). Patients who were younger than 16 years old or with cancer, HIV, or end-stage disease, received immunosuppressive medications, refused to give informed consent to participate in the study, or died within 48 h of ICU admission were excluded from the study. The study was approved by the University of Medicine and Pharmacy at Ho Chi Minh City’s Ethics Committee (reference number 103/ĐHYD-HĐĐĐ). Written informed consent was obtained from either the patients or their legal surrogates when the patients’ health condition prevented them from providing informed consent.

A questionnaire was used to collect baseline information of study participants at ICU admission including demographic characteristics (age, sex, and BMI), comorbidities, clinical signs (blood pressure, respiratory rate, and pulse rate), SOFA and APACHE II scores (27, 28), sepsis stages [sepsis and septic shock (1)], laboratory tests and treatment outcomes. Laboratory tests consisted of complete blood count including WBC, coagulation test, serum lactate, PCT, and the nCD64 index. Regarding nCD64, in addition to the baseline measurement at admission (T0), it was re-measured 48 h thereafter (T48).

### Measurement of the nCD64 index

Given that the flow cytometer is the currently most used method for quantifying nCD64 (17, 18), the flow cytometry BD FACS CANTO system (Becton Dickinson, San Jose, California, United States) was used to perform measurement of the nCD64 index based on regular equipment calibrations with a phycoerythrin (PE) fluorescence quantification kit (Quanti BRITE PE, Becton Dickinson) (29). Fifty μL of EDTA anticoagulated whole blood sample was collected and incubated with 5 μL anti-CD14-FITC (clone MφP9), 5 μL anti-CD64-PE (clone MD22), and 5 μL CD45PerCP (clone 2D1) at room temperature in the dark for 30 min. After lysis, all blood samples were washed and fixed using the BD Lyse/Wash Assistant. The flow cytometer settings were prepared in accordance with the manufacturer’s instructions (29). CD45/CD33-gating was used to isolate monocytes, neutrophils, and lymphocytes, and the median fluorescence intensity (MFI) of CD64 on the cells was measured. The inter-assay standardization for nCD64 quantitation was performed using Quanti BRITE PE calibration beads with known numbers of PE molecules. The MFI values for nCD64 were converted into molecules bound per cell using the BD FACS Diva software (version 6.1.3). This process has been validated elsewhere (30, 31). All laboratory tests were performed at the standardized Laboratory Department of CRH.

Our data collectors, who are qualified nurses with a Bachelor of Nursing, were consistently trained prior to the collection of blood samples. To prevent measurement errors due to prolonged storage of blood samples, nCD64 measurements were performed no later than 4 h post venipuncture as per previously studies (32, 33). In addition, given that the calibrators can be an error source caused by possible lot-to-lot variations and instability (34, 35), the internal quality control is performed daily at the Laboratory Department of our study clinic.

### Sample size calculation

The total sample size was calculated using the formula based on the ROC curves (36). A previous study by Nguyen et al. (37) showed that the survival to death ratio in septic patients at CRH’s ICU was 2:1. In addition, Djordjevic et al. (21) found that the AUC of nCD64 in predicting mortality was 0.727. Hence, using MedCalc version 20.305 (38) with a power of 90%, type I error of 0.05, and default null hypothesis value of 0.5 (i.e., the default null hypothesis is that the AUC is ≤0.5), the minimum total sample size was 72 with a minimum number of survivors of 48 and a minimum number of non-survivors of 24.

For the purposes of our study, participants were classified into two groups including those with sepsis (without shock) and those with septic shock at the time of admission to examine associations between nCD64 and the severity of sepsis. Similarly, to examine the mortality predictive value of nCD64, study participants were also grouped into two other groups including survivors and non-survivors.

### Statistical analysis

The R Statistical Software (version 3.6.2)^1^ was used to perform all statistical analyzes. To examine the performance of the changes in nCD64 values over time in predicting mortality among septic patients and to enable a quick application of nCD64 in busy clinical settings like ICUs, delta nCD64 (i.e., nCD64 T48 – nCD64 T0) and %delta nCD64 [i.e., (nCD64 T48 – nCD64 T0)/nCD64 T0 x 100%] were calculated as suggested elsewhere (39, 40). Categorical data were presented as frequencies and percentages and were analyzed using Pearson’s chi-square test. Normality of distributions of continuous data was examined using Shapiro–Wilk test. Normally distributed continuous data were presented as mean ± standard deviation (SD) and were analyzed using Student’s t test. Non-normally distributed data were presented as median (25th–75th percentile) and were analyzed using Mann–Whitney U test and Spearman’s correlation. The prognostic performance for mortality of the nCD64 index was evaluated using ROC curves. Sensitivity (Sens), specificity (Spec), positive predictive value (PPV) and negative predictive value (NPV) were calculated. The best cutoff values were identified based on maximized Youden’s index [J = max (sensitivity + specificity − 1)]. Survival curves representing mortality were constructed according to the Kaplan–Meier method and compared with the Mantel–Haenszel log-rank test. *p* values <0.05 was considered to be statistically significant.

## Results

### Demographic, clinical, and laboratory characteristics, and treatment outcomes of study participants

**Figure 1:**
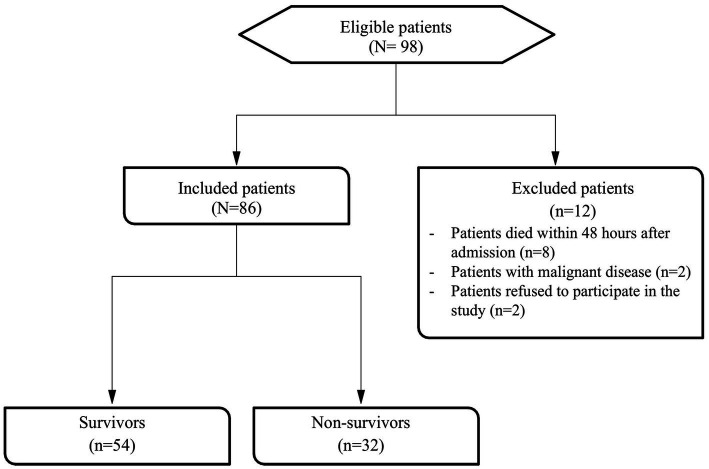
Flowchart of study participants.

Among all 98 septic patients receiving treatment at the study clinic during the study period, 96 agreed to participate in the study making the recruitment rate of 98% (Figure 1). Of these 96 patients, 10 were excluded based on the exclusion criteria. Thus, a total of 86 study participants completed the study and included 54 (63%) survivors and 32 (37%) non-survivors.

Among 86 study participants, the mean age was 60.5 ± 16.3 years, the mean BMI was 23.1 ± 3.2, and male accounted for 45.3% (Table 1). These 86 patients had a median PCT of 34.46 [4.95; 87.33] ng/mL, median ventilator days of 6.5 [4.0; 11.0] and median ICU length of stay of 7.0 [4.0; 13.0] days. Compared with the survivor group, the non-survivor group had significantly higher mean SOFA scores (11.3 ± 4.3 vs. 8.43 ± 3.56, *p* = 0.003), higher median APACHE II scores [24.0 (17.25; 29.0) vs. 19.0 (16.0; 22.25), *p* = 0.003], and higher median serum lactate [3.20 (2.33; 8.03) mmol/l vs. 1.95 (1.38; 3.5) mmol/l, *p* = 0.008] but had significantly lower WBC [11.9 (7.0; 15.1) 10^9/L vs. 15.8 (11.9; 21.1) 10^9/L, *p* = 0.023] and lower hospital length of stay [9.0 (6.25; 18.25) days vs. 19.0 (11.8; 30.25) days, *p* = 0.012]. Regarding nCD64, at T0, there was no statistically significant difference in the nCD64 index between the two groups [3,331 (2,361; 5,634) molecules/cell vs. 3,115 (1,851; 5,388) molecules/cell, *p* = 0.473]. However, at T48, the median nCD64 levels in the survivor group were statistically lower than those in the non-survivor group [2,129 (1,226; 3,503) molecules/cell vs. 2,778 (1,770; 4,983) molecules/cell, *p* = 0.041]. Therefore, compared with the non-survivor group, the survivor group had a significantly lower delta nCD64 [−1,243(−2,702; −231) molecules/cell vs. 234 (−1,328; 922) molecules/cell, *p* = 0.006] and lower %delta nCD64 [−35.3 (−60.1; −10) % vs. 8.0 (−32.7; 45.5) %, *p* = 0.002].

**Table 1:** Demographic, clinical, and laboratory characteristics and treatment outcomes of 86 study participants.

Characteristics	Survivor group* (*N* = 54)	Non-survivor group* (*N* = 32)	Total population* (*N* = 86)	OR	95%CI	*P*
Age (years)	59.4 ± 17.6	62.5 ± 13.9	60.5 ± 16.3	1.01	0.98–1.04	0.386^t^
Male	22 (40.7)	17 (53.1)	39 (45.3)	1.65	0.68–3.98	0.266^c^
BMI (kg/m^2^)	23.2 ± 3.40	23.0 ± 2.88	23.1 ± 3.20	0.99	0.86–1.13	0.836^t^
APACHE II (score)	19.0 [16.0; 22.25]	24.0 [17.25; 29.0]	20.0 [17.0; 25.0]	1.13	1.04–1.23	**0.003**^**u**^
SOFA (score)	8.43 ± 3.56	11.3 ± 4.30	9.49 ± 4.07	1.21	1.07–1.37	**0.003**^**t**^
Serum lactate (mmol/l)	1.95 [1.38; 3.5]	3.20 [2.33; 8.03]	2.70 [1.49; 4.43]	1.29	1.07–1.55	**0.008**^**u**^
PCT (ng/mL)	27.18 [5.53; 100.78]	44.8 [2.87; 79.93]	34.46 [4.95; 87.33]	1.00	0.99–1.00	0.517^u^
WBC (10^9/L)	15.8 [11.9; 21.1]	11.9 [7.0; 15.1]	14.0 [9.8; 19.0]	0.93	0.87–0.99	**0.023**^**u**^
nCD64 T0	3,331 [2,361; 5,634]	3,115 [1,851; 5,388]	3,205 [2,209; 5,620]	1.00	1.00–1.00	0.473^u^
nCD64 T48	2,129 [1,226; 3,503]	2,778 [1,770; 4,983]	2,284 [1,362; 4,279]	1.0002	1.00001–1.0004	**0.041**^**u**^
delta nCD64	-1,243 [−2,702; −231]	234 [−1,328; 922]	−640 [−2,047; 390]	1.0003	1.0001–1.0006	**0.006**^**u**^
%delta nCD64	−35.3 [−60.1; −10]	8.0 [−32.7; 45.5]	−27.1 [−55.7; 17.6]	4.96	1.82–13.50	**0.002**^**u**^
Ventilator days (days)	6.0 [3.0; 9.25]	8.0 [5.0; 13.5]	6.5 [4.0;11.0]	1.03	0.98–1.09	0.182^u^
ICU length of stay (days)	7.0 [4.0; 11.5]	8.0 [4.0; 14.0]	7.0 [4.0;13.0]	1.01	0.96–1.06	0.718^u^
Hospital length of stay (days)	19.0 [11.8; 30.25]	9.0 [6.25; 18.25]	14.5 [9.0; 24.0]	0.94	0.90–0.99	**0.012**^**u**^

The bold *p* value means the associated analysis is statistically significant.BMI, Body mass index; APACHE, Acute Physiology and Chronic Health Evaluation; SOFA, sequential organ failure assessment score; ICU, intensive care unit.*Continuous data are presented as mean ± (standard deviation) or median [25th–75th percentile]; categorical data were presented as an absolute count (percentage).^t^*t*-test.^u^Mann-Whitney U test.^c^Pearson Chi square test.

### Comparison of nCD64 index at T0 with APACHE II and SOFA score between patients with sepsis and those with septic shock

Among 86 patients with sepsis, 32 (37%) had septic shock (Table 2). There was a statistically significant difference between the septic and septic shock group regarding the median APACHE II scores, SOFA scores, and nCD64 values T0 (*p* < 0.05).

**Table 2:** Distributions of APACHE II and SOFA scores and laboratory parameters in the sepsis and septic shock groups.

Characteristics	Sepsis group* *N* = 54	Septic shock group* *N* = 32	*P*^u^
APACHE II (score)	15.5 [12.25; 21.0]	21.0 [17.5; 45.0]	**0.001**
SOFA (score)	5.0 [3.0; 6.0]	10.0 [8.0; 12.5]	**<0.001**
nCD64 T0	1,514 [1,416; 2,542]	3,568 [2,589; 5,999]	**<0.001**

The bold *p* value means the associated analysis is statistically significant.*Data are presented as median [25th–75th percentile].^u^Mann-Whitney U test.

**Figure 2:**
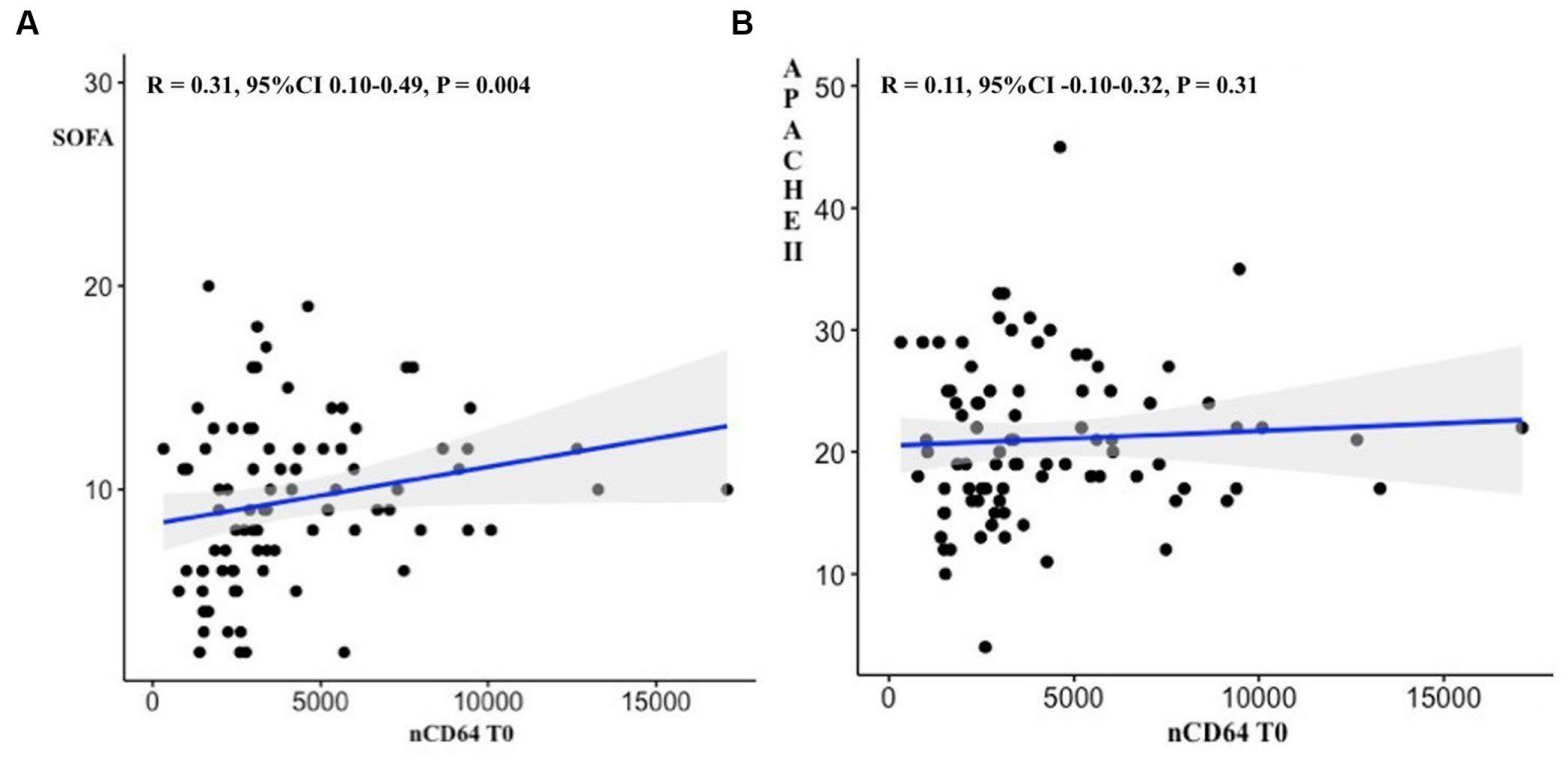
Correlations between nCD64 values at ICU admission and SOFA scores **(A)** as well as APHACHEII scores **(B)**.

There was a medium positive linear correlation between nCD64 values T0 and SOFA scores (*R* = 0.31, 95%CI 0.10–0.49, *p* = 0.004) but not between nCD64 T0 values and APACHE II scores (*R* = 0.11, 95%CI -0.10-0.32, *p* = 0.31; Figure 2).

### Dynamic of nCD64 in the first 48 h between survivor and non-survivor groups

**Figure 3:**
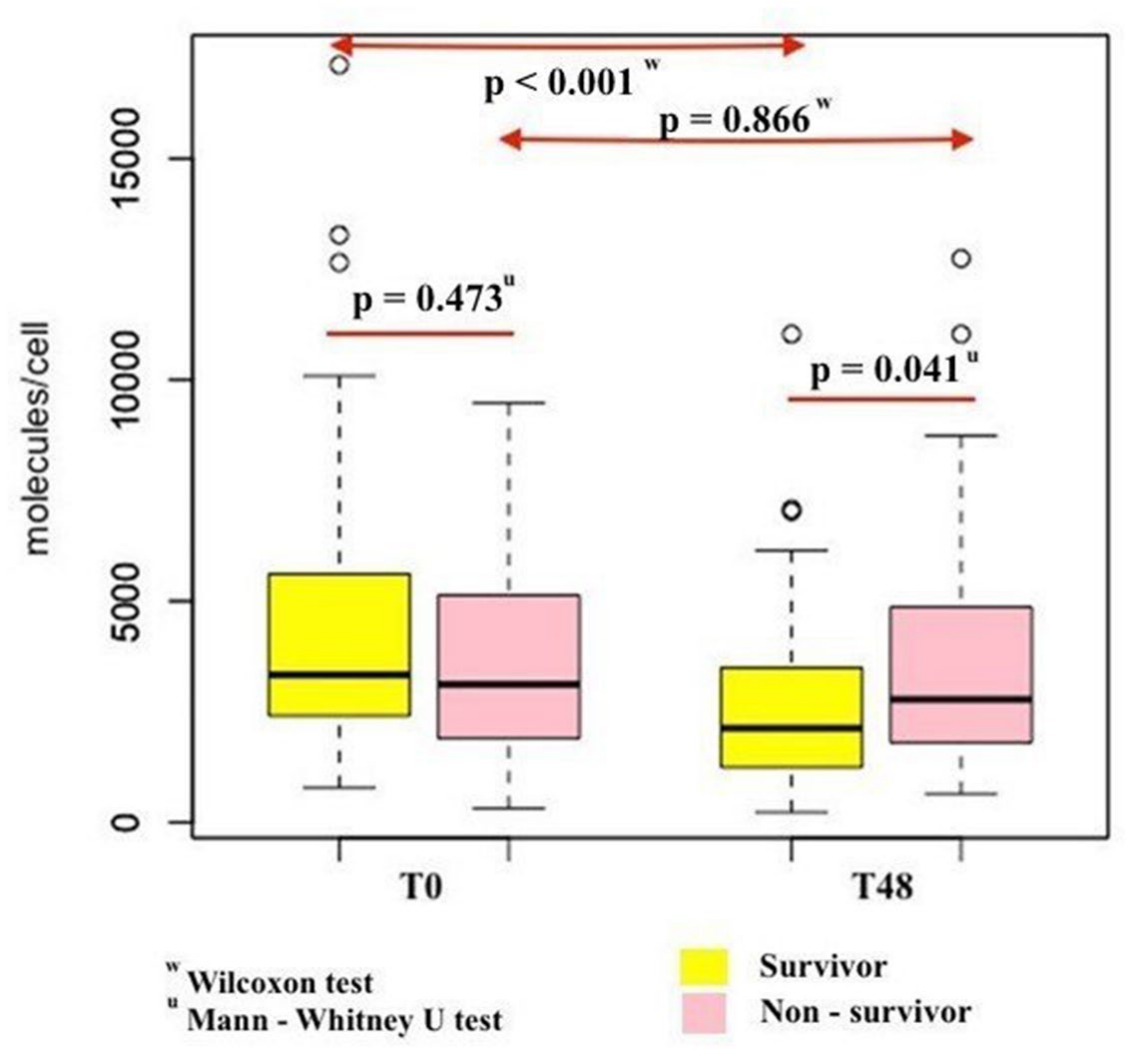
Comparison of changes in nCD64 levels over the first 48 h after ICU admission between survivor and non-survivor patients.

**Figure 4:**
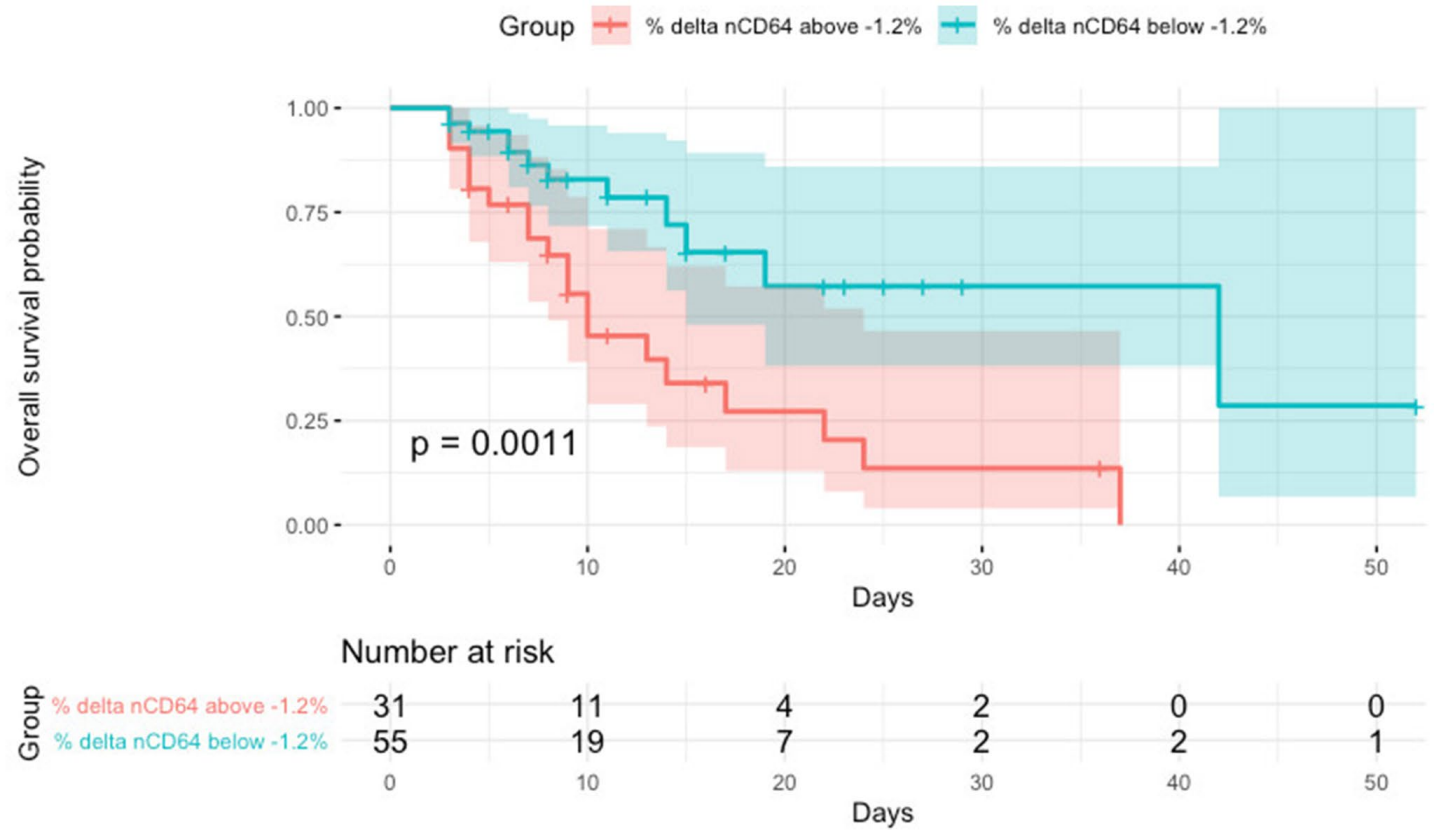
Kaplan–Meier overall survival analysis of septic patients with regard to the changes in nCD64 over time (%delta nCD64).

The nCD64 levels decreased significantly over the first 48 h after ICU admission in the survivor group (*p* < 0.001), but this change was not statistically significant in the non-survivor group (*p* = 0.866; Figure 3). The survival of patients with %delta nCD64 below −1.2% was significantly higher compared to those with %delta nCD64 above −1.2% (*p* = 0.0011; Figure 4).

### Predictive values of nCD64, SOFA and APACHE II scores and combinations of these parameters

**Figure 5:**
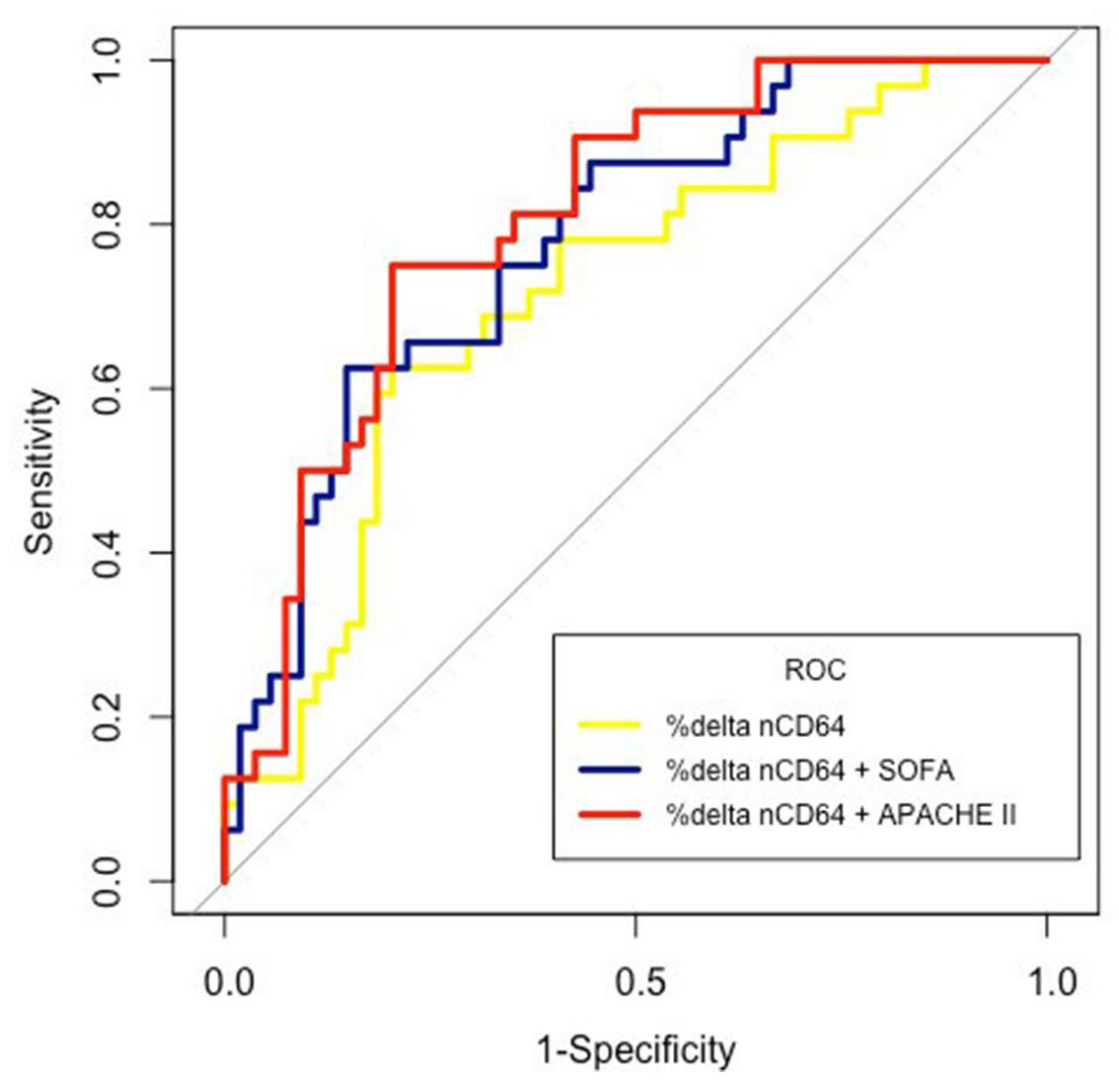
ROC curves comparing ICU mortality prognostic abilities of %delta nCD64 and %delta nCD64 combined with SOFA score as well as APACHE II scores.

The AUC values of %delta nCD64 (0.72) demonstrated a greater level of accuracy compared to delta nCD64 (0.7), SOFA score (0.7), APACHE II score (0.68), nCD64 T48 (0.63), and nCD64 T0 (0.53; Table 3; Figure 5). The highest AUC was achieved when %delta nCD64 was combined with the APACHE II score (0.81), which was comparable to the AUC of %delta nCD64 combined with both the APACHE II and SOFA scores (0.81). By analyzing the ROC curve using %delta nCD64 in combination with the APACHE II score, these combined parameters exhibited the ability to predict mortality with 75% sensitivity, 79.6% specificity, 68.6% PPV, and 84.3% NPV. Similarly, based on the ROC curve of using %delta nCD64 in conjunction with both the APACHE II and SOFA scores, these combined parameters displayed the capacity to predict mortality with 78.1% sensitivity, 75.9% specificity, 65.8% PPV, and 85.4% NPV.

**Table 3:** ICU mortality prognostic values of nCD64, SOFA and APACHE II scores among 86 study participants.

Biomarkers	AUC	95%CI	Cut-off	Sens	Spec	PPV	NPV
nCD64 T0	0.53	0.40–0.66	<2,390	37.5%	75.9%	48.0%	67.2%
nCD64 T48	0.63	0.51–0.75	>1,294	93.8%	27.8%	43.5%	88.2%
SOFA score	0.70	0.58–0.65	>9.5	68.8%	63.0%	52.4%	77.3%
APACHE II score	0.68	0.55–0.81	>26	43.8%	92.6%	77.8%	73.5%
Delta nCD64	0.70	0.58–0.81	> − 49	62.5%	79.6%	64.5%	78.2%
% delta nCD64	0.72	0.60–0.83	> − 1.2	62.5%	79.6%	64.5%	78.2%
% delta nCD64 + APACHE II	0.81	0.71–0.90		75.0%	79.6%	68.6%	84.3%
% delta nCD64 + SOFA	0.78	0.68–0.88		62.5%	85.2%	71.4%	79.3%
% delta nCD64 + APACHE II + SOFA	0.81	0.72–0.90		78.1%	75.9%	65.8%	85.4%

AUC, area under the curve; Sens, sensitivity; Spec, specificity; PPV, positive predictive value; NPV, negative predictive value.

## Discussion

We documented a mortality rate of 37.2% among septic patients, which is similar to the rates reported in previous studies (4, 37). In detail, a study conducted at the same ICU in 2017 reported a mortality rate of 33.3% (13/39, 95%CI 20.6–49.0%) (37). Another study conducted in 2022 at ICUs located across 22 Asian countries found that in settings that are comparable to our study context, the mortality rate was 35.5% (196/552, 95%CI 31.6–39.6%) (4). We also found that the APACHE II and SOFA scores as well as serum lactate levels of the non-survivor group were statistically higher than those of the survivor group. This reflects greater levels of severity in deceased patients through previously proven severity scores (10, 25). Similarly, several studies have shown that higher serum lactate levels are associated with an increased sepsis mortality (41, 42). Besides, in our study, the deceased group had significantly lower WBC counts than the survivor group. Indeed, a large retrospective study of 5,909 ICU patients has found that leukopenia is associated with a higher risk of mortality compared to leukocytosis (OR = 1.6, 95%CI 1.2–2.2) after adjusting for demographic characteristics and comorbidities using variates analogous to those used for sepsis-3 criteria (43). In addition, we found that the deceased group had a significantly shorter hospital length of stay than the survivor group, which may be owing to the deceased group’s severe condition leading to early mortality and thus, shorter hospital length of stay.

CD64 is a protein found on the surface of certain immune cells, such as neutrophils and monocytes (12). nCD64 expression increases once these neutrophils are stimulated by the proinflammatory cytokines granulocyte colony-stimulating factor (G-CSF) and interferon gamma (IFN-γ), which are produced in response to infection or after exposure to endotoxin (32). Therefore, the role of nCD64 in the early diagnosis of sepsis has been well reported (16, 44, 45). However, the use of nCD64 as a prognostic indicator in critically ill patients has received little attention (18). We found that the nCD64 index was associated with sepsis stages. Patients with septic shock had significantly higher nCD64 levels compared with septic patients. Our result is consistent with other available nCD64 studies (14, 32, 46, 47). In a prospective study by Hsu et al., nCD64 expression has been found to increase progressively from patients with systemic inflammatory response syndrome (SIRS) to severe sepsis and septic shock (46). It has also been well documented that nCD64 is better than PCT in differentiating SIRS from severe sepsis and septic shock (46). Similarly, Ghosh et al. have found that nCD64 levels are significantly higher in patients with septic shock compared with septic patients on days 0 (47). In a more recent study using sepsis-3 criteria like us, Yin et al. have also found that nCD64 levels increase significantly with the disease severity. Patients with septic shock exhibit higher levels of nCD64 expression compared to those with a less severe infection (14). Like another study (32), the correlations between stages of sepsis and nCD64 values documented 48 h after ICU admission as well as the changes in nCD64 values over time (i.e., delta nCD64 and %delta nCD64) were not examined in our study. This is because in clinical practice, confirming the progress of sepsis is usually important at the time when patients are admitted to the ICU. After 48 h post ICU admission, the sepsis stages are clear, and the patient’s mortality prognosis has become an important concern (48). A tool commonly used to assess the severity of sepsis is the SOFA score, which is a clinical scoring system based on an evaluation of the function of different organ systems in critically ill patients (10). In our study, we found that nCD64 levels were positively associated with SOFA scores. Indeed, it has been found that an increase in nCD64 levels due to an activation of CD64 expression in neutrophils by bacterial infection reflects the disease severity (12). Like us, several studies have shown a positive correlation between nCD64 values and SOFA scores in septic patients, suggesting that higher levels of nCD64 are associated with higher SOFA scores and worse outcomes (49–51). The nCD64 index has also been found to be more closely related to the severity of sepsis than other biomarkers including PCT, CRP, IL-6, and IL-10 (52). Considering these findings, we strongly believe that the nCD64 index can be used as a valuable biomarker in estimating the severity of sepsis.

The ability of nCD64 to predict sepsis-related mortality remains controversial. A prospective study conducted on 132 ICU patients in Spain has found that septic patients who survive had a higher nCD64 index compared with deceased patients (20). This may be due to the “exhaustion” of neutrophils brought on by constant stimulation from systemic cytokines in non-survivors (20). Another prospective study conducted on 41 septic patients in an ICU in Greece has found that those with a lower and higher nCD64 index have a worse and better outcome, respectively (19). Therefore, the reduced neutrophil phagocytic activity during the first 24 h after ICU admission is a predictor of mortality (19). In contrast, other studies have showed a negative correlation between nCD64 levels and survival. Djordjevic et al. have reported that the nCD64 values on day 1 and day 2 post admission are higher in non-survivors compared with survivors (21). Especially, the difference in the nCD64 values between these two groups is more pronounced on day 2 than day 1 (21). Similarly, in a recent study with 349 septic patients, Huang et al. have showed that non-survivor patients have a higher nCD64 index than survivors (22). In another study assessing serial nCD64 measurements in septic patients over the first 8 days of ICU stay, Ghosh et al. have found that there is no significant difference in the nCD64 index on days 0 and 4 between survivor and deceased group, but the nCD64 index in deceased patients is higher on day 8 (47). In our study, the nCD64 index on day 0 (T0) was not significantly different between the two groups, but this parameter on day 2 (T48) was higher in non-survivor group compared with survivors. In addition, ROC curve analysis of the nCD64 index at T48 in predicting mortality showed an AUC of 0.63. In light of our findings, a follow-up examination of the nCD64 index at day 2 post ICU admission would enhance its accuracy in predicting mortality in septic patients.

Few studies have been conducted to examine the changes in nCD64 levels over time in relation to the prediction of mortality in septic patients (18). The clinical status of critically ill septic patients may alter in the first few days of ICU admission due to resuscitation, antibiotic use, and other treatments (18). Hence, it has been documented that when a biomarker is used for its prognostic utility in ICU settings, a serial analysis of its values over time is more reliable than a single value at admission (18). Concurring with this, we found that the AUC value in predicting ICU mortality of delta nCD64 and %delta nCD64 were 0.7 and 0.72, respectively, demonstrating a high capacity in predicting mortality of serial nCD64 examination in the first 48 h after ICU admission. The AUC of %delta nCD64 was also better than that of SOFA score and APACHE II score. We found that nCD64 values decreased significantly in the survivor group. This means the higher the value of %delta nCD64, the higher risk of mortality in septic patients. Like us, in a prospective observational study of 155 patients with longitudinal course of nCD64, De Jong et al. have found a more substantial decrease in the mean nCD64 index over time in the survivor group (53). The authors also note a decline in nCD64 values after day 3, which could be explained by either an indirect effect of antibiotics inducing restoration of the regulated immune response or a condition of neutrophil deactivation with a decreased polymorphonuclear neutrophils phagocytic function (53). Similarly, Ghosh et al. have reported that survivors have a significant decrease in nCD64 values over time (47). In a more recent study, Cui et al. have also documented an inverse association between a decline in the serial nCD64 index and in-hospital death rates (54). In light of these studies and our findings, we believe that monitoring nCD64 levels over time could be a useful tool to predict mortality in septic patients.

Given that sepsis is a highly complex immunological syndrome involving both hyperinflammation and immunosuppression (7), a single biomarker may not be able to provide a comprehensive understanding of a patient’s immune status. Thus, combining different markers has been suggested (22). Regarding the use of nCD64, in a systematic review of all published studies between 2006 and 2019, Patnaik et al. have also recommended that nCD64 should be utilized in a combination with other sepsis biomarkers for prognosis in critically ill patients, and the kinetics of serial nCD64 trend is helpful in examining different aspects of prediction (18). However, to the best of our knowledge, till now, there have only been a few studies examining nCD64 in combination with other parameters for sepsis prognostication (14, 22, 55). Qiqi Chen et al. have found that nCD64 combined with the APACHE II score has a significantly higher level of accuracy in mortality prediction compared with separate uses of these parameters (55). Yin et al. have also found that the AUC of nCD64 or PCT combined with the SOFA score is significantly higher than that of any single measure for predicting 28-day mortality in septic patients in ICU settings (14). Similarly, Huang et al. have proved that nCD64 plus CRP have a better performance in the prediction, discrimination, and reclassification of the 28-day mortality risk in septic patients (22). Although we did not examine CRP due to our study clinic’s policy, we also found that the AUC of %delta nCD64 combined with the APACHE II score (0.81) was higher than that of any other parameter alone or in combination with each other, except %delta nCD64 combined with both APACHE II and SOFA scores which was also 0.81. Therefore, given an ICU setting, to improve the predictive efficacy in septic patients, we believe that the best approach is to examine the changes in nCD64 over time in combination with using the APACHE II score.

Our study has some limitations. First, given the single-center study design, the generalizability of our data may be limited to comparable settings. Second, monitoring the nCD64 index for a longer period than 48 h may make the predictive values of this biomarker more obvious. Unfortunately, we were unable to do this due to limited resources. Third, although data on the differential leukocyte count and immunoglobulins levels provide more insight into understanding the association between the dynamic of nCD64 over time and the course of sepsis, these data were not examined in our study. Fourth, despite the performance of sample size calculation, the actual sample size is considerably small. Given that sepsis is a prevalent health condition, we believe studies with larger sample size could provide more robust and generalizable results. Our study was based on delta nCD64 and %delta nCD64 as measures to examine the performance of changes in nCD64 values over time in predicting mortality. Although the dynamic of nCD64 over time has been examined in several similar studies (32, 47, 53, 54), the methods used to quantify these changes in nCD64 have not been standardized. To the best of our knowledge, however, this is the first study in Vietnam and among the few studies worldwide to evaluate the performance of the nCD64 index in predicting mortality in Asian patients with sepsis. In addition, our study is among the first ones that used the most recent definition of sepsis (sepsis-3 criteria) in examining nCD64 (1).

## Conclusion

The nCD64 index could be a reliable biomarker to predict the progress of sepsis including mortality. Monitoring the kinetics of serial nCD64 trend during the first 2 days of ICU stay is helpful in predicting the outcome of septic patients. The use of a combination of the trends of nCD64 index with the APACHE II score would further enhance the predictive accuracy. More studies with longer follow-ups are needed to fully understand the implications of serial trend and kinetics of nCD64 in septic patients including the prediction of the long-term morbidity and quality of life post-sepsis. To assist in clinical decision making, it is also important to explore more reliable parameters that help quantify the absolute risks of severe sepsis and sepsis-related mortality.

## Data availability statement

The raw data supporting the conclusions of this article will be made available by the authors, without undue reservation.

## Ethics statement

The studies involving humans were approved by University of Medicine and Pharmacy at Ho Chi Minh City (approval number 103/ĐHYD-HĐĐĐ). The studies were conducted in accordance with the local legislation and institutional requirements. The participants provided their written informed consent to participate in this study.

## Author contributions

HP, DN, MD, LT, DT, XP, and TP designed the study, performed statistical analysis and drafted the manuscript. HP, DN, LT, DT, XP, and TP performed data collection. All authors contributed to the article and approved the submitted version.

## Conflict of interest

The authors declare that the research was conducted in the absence of any commercial or financial relationships that could be construed as a potential conflict of interest.

## Publisher’s note

All claims expressed in this article are solely those of the authors and do not necessarily represent those of their affiliated organizations, or those of the publisher, the editors and the reviewers. Any product that may be evaluated in this article, or claim that may be made by its manufacturer, is not guaranteed or endorsed by the publisher.

## Footnotes

^1^http://www.R-project.org

## Abbreviations

APACHE: Acute Physiology and Chronic Health Evaluation
AUC: Area under the ROC curve
CRH: Cho Ray Hospital
CRP: C reactive protein
FcγR1: Fc-receptor I
G-CSF: Granulocyte colony-stimulating factor
IFN-γ: Interferon gamma
ICUs: Intensive care units
MFI: Median fluorescence intensity
NPV: Negative predictive value
nCD64: Neutrophil cluster of differentiation 64
PE: Phycoerythrin
PPV: Positive predictive value
PCT: Procalcitonin
ROC: Receiver operating characteristic
Sens: Sensitivity
SOFA: Sequential Organ Failure Assessment
Spec: Specificity
SIRS: Systemic inflammatory response syndrome
Sepsis-3: Third International Consensus Definitions for Sepsis and Septic Shock
WBC: White blood cell
